# Supplementary material for: Multiagent Intratumoral Immunotherapy Can Be Effective in A20 Lymphoma Clearance and Generation of Systemic T Cell Immunity
Source: Cancers (Basel). 2023 Mar 24;15(7):1951. doi: 10.3390/cancers15071951 (PMC10093573; doi:10.3390/cancers15071951)
Supplement: Supplementary file 1 [file cancers-15-01951-s001.zip › cancers-2230441-supplementary.pdf]

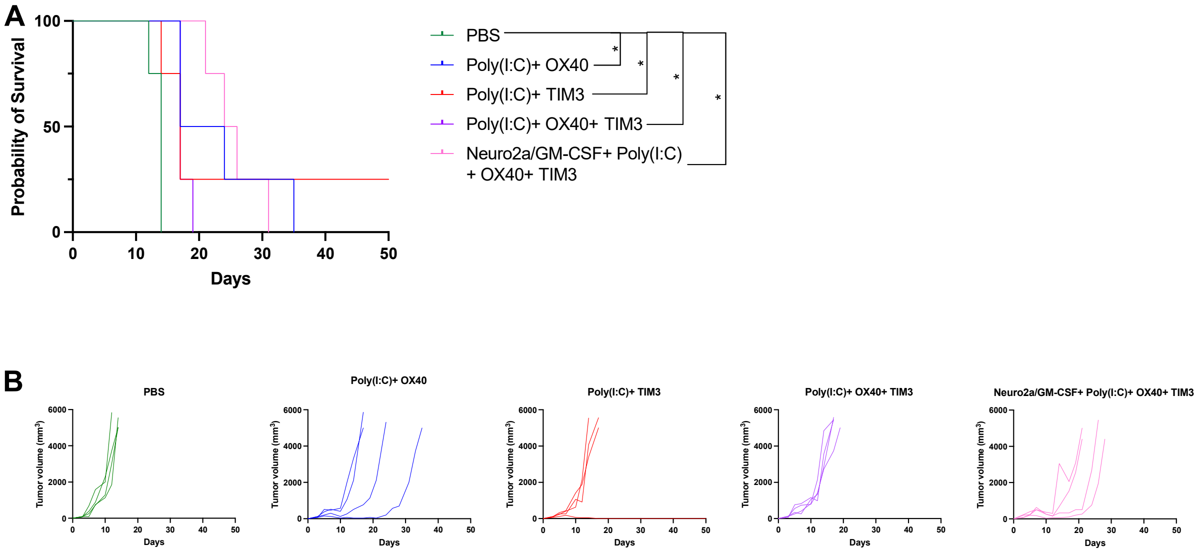

Figure S1. *Combination immunotherapy slows B16 tumor growth and modestly improves survival.* Mice were randomized into treatment groups (n=4/group) and received 3 doses of indicated treatments, as described in Section 2. **(A)** Kaplan-Meier survival curves of each treatment group with statistical differences assessed via the Log rank test: **P<0.05*. **(B)** Individual mouse tumor growth by treatment group. Tumors were sized 3 times per week and tumor size was calculated as described in Section 2. Mice were removed from the study once tumors reached a size of >5000mm³.

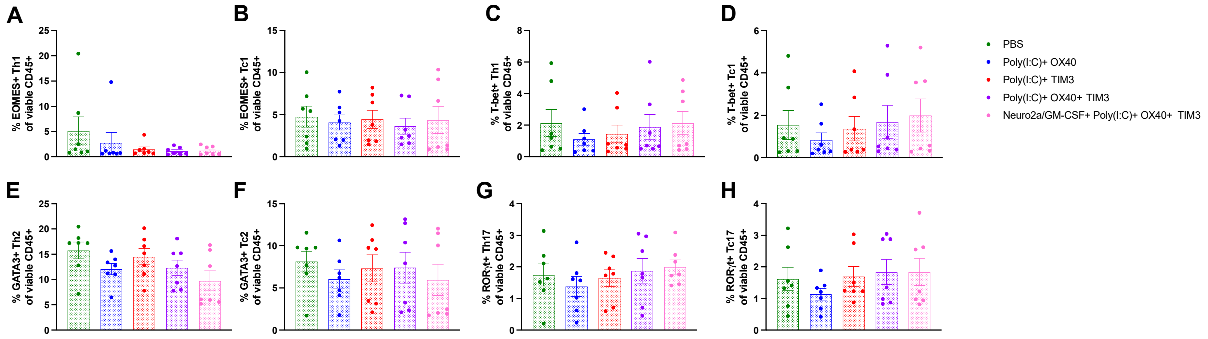

Figure S2. *Splenic T cell polarization is variable following intratumoral immunotherapy regimens.* Spleens were harvested 24 hours following the second immunotherapy dose and prepared for flow cytometric analysis. T cell polarization was identified via specific transcription factors and are shown as a percentage of total viable CD45+ cells and gated as follows: **(A)** CD4+EOMES+ Th1 **(B)** CD8+EOMES+ Tc1 **(C)** CD4+T-bet+ Th1 **(D)** CD8+T-bet+ Tc1 **(E)** CD4+GATA3+ Th2 **(F)** CD8+GATA3+ Tc2 **(G)** CD4+RORγt+ Th17 **(H)** CD8+RORγt+ Tc17 **(A-H)** Statistical differences were determined with a one-way ANOVA followed by Tukey's post-hoc comparisons. No groups were significantly different from each other.

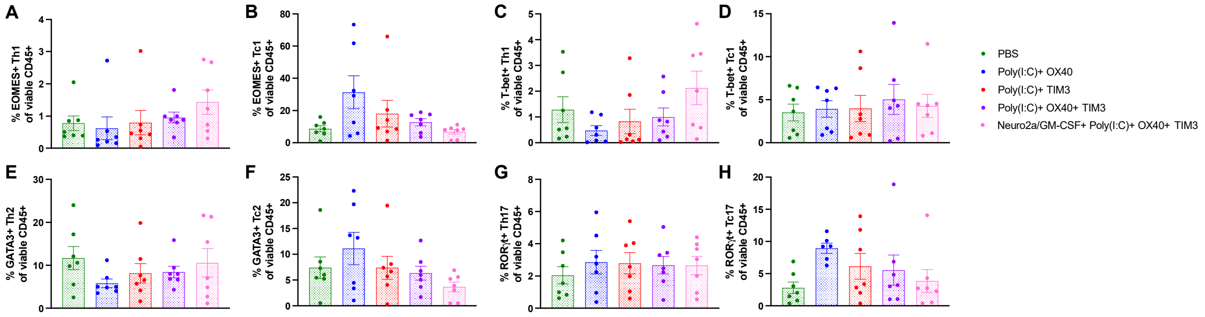

Figure S3. *Tumor T cell polarization is variable at 24 hours post-intratumoral immunotherapy.* Tumors were harvested 24 hours following the second immunotherapy dose and prepared for flow cytometric analysis. T cell polarization was identified via specific transcription factors and are shown as a percentage of total viable CD45+ cells and gated as follows: **(A)** CD4+EOMES+ Th1 **(B)** CD8+EOMES+ Tc1 **(C)** CD4+T-bet+ Th1 **(D)** CD8+T-bet+ Tc1 **(E)** CD4+GATA3+ Th2 **(F)** CD8+GATA3+ Tc2 **(G)** CD4+RORγt+ Th17 **(H)** CD8+RORγt+ Tc17 **(A-H)** Statistical differences were determined with a one-way ANOVA followed by Tukey's post-hoc comparisons. No groups were significantly different from each other.

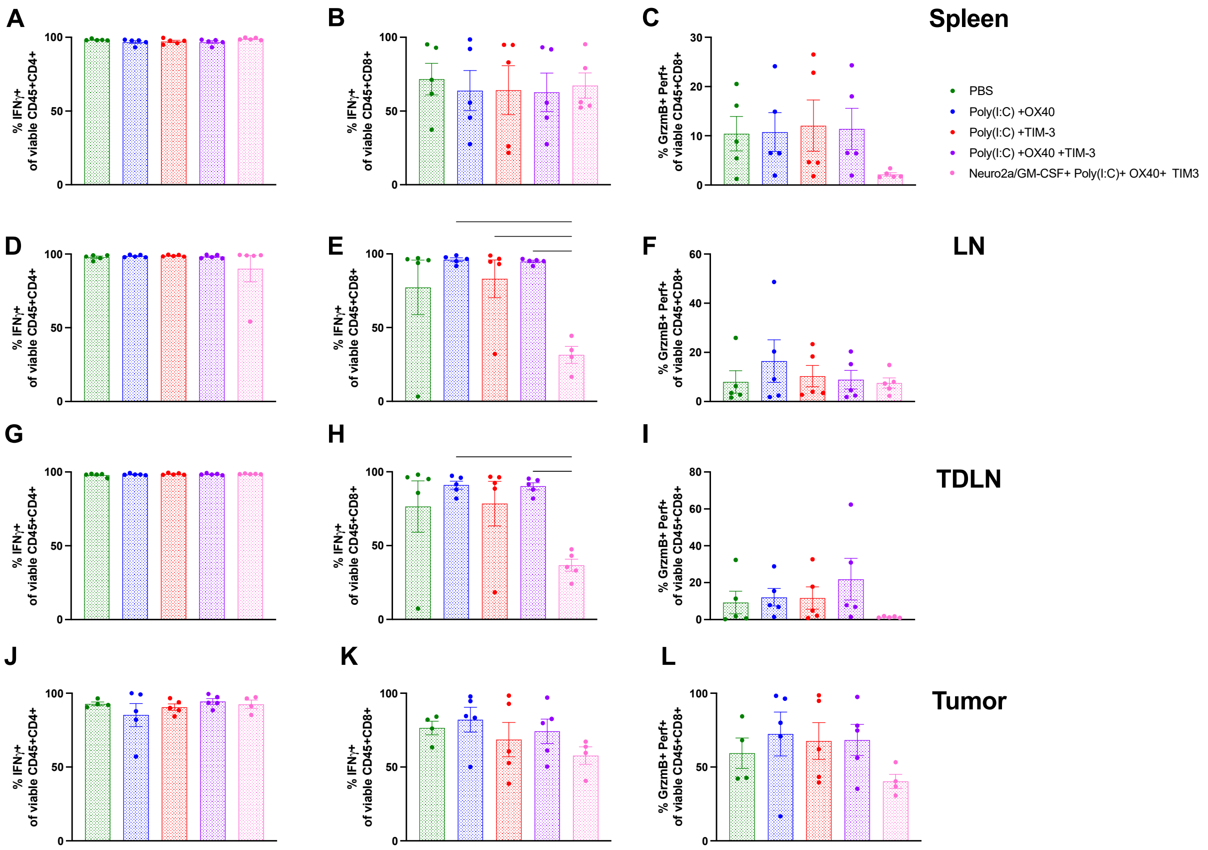

Figure S4. Markers of T cell functionality of total CD4 or CD8 T cells modestly altered post-immunotherapy. **(A-C)** Spleens, **(D-F)** lymph nodes (LN), **(G-I)** tumor-draining lymph nodes (TDLN), and **(J-L)** tumors were harvested 24 hours after the second immunotherapy dose and prepared for flow cytometric analysis as described in Section 2. All groups are gated from viable CD45+ cells and are presented as a percentage of either total CD4 or CD8 cells, indicated on graphs. Pro-inflammatory T cells were identified via the presence of interferon-γ (IFN-γ) in **(A, D, G, J)** CD4+ T cells or **(B, E, H, K)** CD8+ T cells. **(C, F, I, L)** Active cytotoxic CD8 T cells were identified via co-expression of Granzyme B (GrzmB) and Perforin (Perf). **(A-L)** Statistical analyses were done using a one-way ANOVA with Tukey's post-hoc test. The level of significance is denoted by the number of asterisks: *P<0.05, **P<0.01.

Table S1. *Antibodies used for flow cytometric analysis.*

Marker	Company	Clone	Catalong no.	Fluor
Viability	BD Bioscience	N/A	65-0868-18	FVD455UV
CD45	Biolegend	30-F11	103122	AF488
CD3	Biolegend	145-2C11	100328	PerCP-Cy5.5
CD4	Biolegend	GK1.5	100438	BV421
CD8a	Biolegend	53-6.7	100766	APC-Fire750
CD62L	Biolegend	MEL-14	104412	APC
CD44	Biolegend	IM7	103059	BV785
Ki67	Biolegend	11F6	151210	PE
CD335/NKp46	Biolegend	29A1.4	137618	PE-Cy7
CD19	Biolegend	6D5	115554	PE-594
CD25	Biolegend	PC61	102010	PE-Cy5
FoxP3	eBiosciences	FJK-16s	12-5773-82	PE
T-bet	BD Bioscience	O4-46	562467	PE-CF594
GATA3	BD Bioscience	L50-823	560405	PE-Cy7
RORgt	BD Bioscience	Q31-378	564723	BV786
EOMES	eBiosciences	Dan11mag	17-4875-82	APC
Granzyme B	eBiosciences	NGZB	50-8898-82	eFluor660
Perforin	eBiosciences	eBioOMAK-D	12-9392-82	PE
IFN-g	Biolegend	XMG1.2	505826	PE-Cy7
CD4	Biolegend	RM4-4	116023	BV421
CD8	Thermo	CT-CD8a	MA5-17599	PE
